# Dynamics and Structure of a Bitumen Emulsion as Studied by ^1^H NMR Diffusometry

**DOI:** 10.1021/acsomega.3c05492

**Published:** 2023-09-21

**Authors:** Andrei Filippov, Hilde Soenen, Johan Blom, Oleg N. Antzutkin

**Affiliations:** † Chemistry of Interfaces, Department of Civil and Environmental Engineering, Luleå University of Technology, Luleå SE-97187, Sweden; ‡ Nynas N.V., 171 Groenenborgerlaan, Antwerp 2020, Belgium; § Faculty of Applied Engineering, EMIB-Research Group, University of Antwerp, 171 Groenenborgerlaan, Antwerp 2020, Belgium

## Abstract

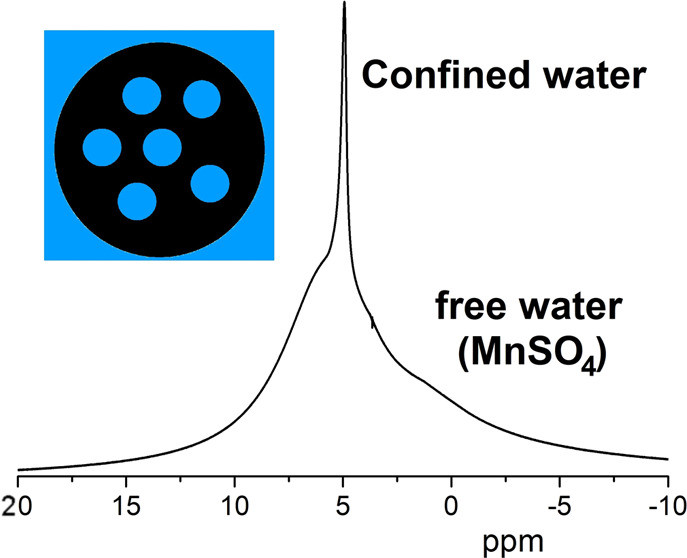

Self-diffusion in a bitumen emulsion was studied by ^1^H NMR. The emulsion forms two phases: continuous and dispersed. The continuous aqueous phase contains mainly water, with the energy of activation of the diffusion process equal to that of bulk water, while its diffusivity is smaller than that of bulk water by a factor of 2. The dispersed phase consists of bitumen droplets containing confined water, whose dynamics is characterized by a fully restricted diffusion regime in cavities with sizes of ∼0.11 μm. Therefore, the studied bitumen emulsion can be described by a model of a complex multiple emulsion of the water/oil/water (WOW) type. The suggested model does agree well with data from ^1^H NMR spectroscopy and diffusometry of the bitumen emulsion doped with paramagnetic MnSO_4_(aq) as well as with an additional ^1^H NMR study of the emulsion structure, in which emulsion stability was compromised by freezing at 253 K.

## Introduction

There is a need to enhance asphalt technology by enabling production at lower temperatures, while not compromising performance compared to asphalt produced by conventional approaches.^1−3^ Bitumen emulsion mixtures used in cold asphalt technology offer certain advantages over hot bituminous road mixtures in terms of potential cost savings, environmental factors, energy savings, and reducing logistical difficulties inherent with a hot mix.^3^

An emulsion is a dispersion of small droplets of one liquid in another liquid, forming dispersed and continuous phases, respectively, due to the immiscibility of the liquids.^4^ In most emulsions, one of the phases is water, and the other water-immiscible phase is “oil”. Therefore, emulsions are “oil-in-water” (O/W) or “water-in-oil” (W/O) where the continuous phase is water or oil, respectively. Multiple emulsions,^5^ so-called water-in-oil-in-water (W/O/W) emulsions consist of water droplets dispersed in oil globules, themselves ultimately dispersed in an aqueous phase. Bitumen emulsions typically contain 40–70% bitumen by weight in the form of droplets 1–20 μm in diameter. Water content ranges from 25 to 60 wt %. Emulsions are inherently unstable. For this reason, to increase the stability of the emulsion, a small amount (0.1–2.0 wt %) of a surfactant (an emulsifier) is usually added. Molecules of the emulsifier typically adsorb on the water–oil interfaces and provide forces that keep the bitumen droplets apart. For some particular applications, the emulsion properties can be modified by adding polymers, acids, salts, and thickeners. There is a distribution of particle sizes (dispersed phase) in emulsions, which may have an influence on the mechanical properties.

Bitumen emulsions are normally considered the O/W type, but several studies have shown that some water is confined within the bitumen droplets,^6−10^ as shown by microscopy^11^ and calorimetry.^8,10,12^ Therefore, such emulsions are best considered as the W/O/W type. The emulsion type affects system properties such as viscosity^6,10^ and thermal behavior.^8,10,12^ The concentration of “internal” water has been estimated to be up to 13 wt %^8^ or, in the other study, up to 10 wt %^10^ of the emulsion. This type of water is confined in larger bitumen droplets,^9^ with water droplet size influenced by the emulsifier and salt content,^10^ and gradually changed with temperature and storage time and conditions.^10^ In spite of the importance of the W/O/W structure for physical and applied properties of bitumen emulsions, no details of the molecular dynamics (including dynamics of the confined water) have been studied to date.

Displacements of molecules in the bulk fluid phase follow Gaussian diffusion.^13^ In the presence of restrictions, the statistics of translational displacements is no longer Gaussian and is usually presented as a spectrum of apparent diffusion coefficients, which is diffusion time (*t*_d_)-dependent (here, *t*_d_ is a specific parameter of the NMR pulse sequence).^13^ Pulsed field gradient (PFG) NMR, also known as NMR diffusometry, is a powerful method for determining the structure of microheterogeneous systems.^13−25^ Primary information about the diffusivity of moving particles is contained in the diffusion decay (DD) of the spin–echo amplitude, which has a single-exponential form in a bulk homogeneous single-component liquid, but it is described by a more complex mathematical form in the presence of spatial restrictions.^17,19^ The effects of a number of simple geometries of restrictions—spheres, cylinders, and planes—on DDs have been analyzed previously.^13,20,21^ The results were applied to the diffusion of the W/O type to estimate the sizes and size distributions of the emulsion droplets.^22,26^ Oscillations of DDs predicted by simulations^22,21^ were observed for emulsions with a narrow size distribution of droplets.^23^

Regarding bitumen emulsions, PFG NMR methods have been applied alongside NMR relaxometry to study the stability of the water-in-oil (W/O)-type bitumen emulsion.^27^ A complicated form of the ^1^H NMR diffusion decay has been analyzed, suggesting restricted diffusion of water in water droplets. A Gaussian distribution of the sizes of the water droplets has been obtained with a mean value of 14 μm.^27^^1^H PFG NMR has also been used to monitor crude oil emulsion separation.^28^

The purpose of this work was to analyze the structure of a certain type of bitumen emulsion used in the cold mix asphalt technology by applying the ^1^H PFG NMR method.

## Materials and Methods

### Bitumen Emulsion

A slow-breaking bitumen emulsion of the EN-grade: C67B4-160/220 (according to EN 13808) was kindly provided by Nynas AB. This type of emulsion in combination with a breaking additive is used in production of the cold mix asphalt.^1,3^ To evaluate the size of the emulsion droplets, a Keyence VK-X1000 confocal laser scanning microscope (CLSM) equipped with a VK-D1 motorized XY stage was employed. The CLSM uses two light sources: a laser light source, in this case with a wavelength of 661 nm, and a white light source. The laser information provides lateral resolution and measurement data, while the white light source enables the capture of color information, similar to an optical microscope. Different magnification levels, from 140× (28 × 5) to 16,800 (4 × 28 × 150), of a Nikon Plan Apo EPI objective lens were available. The lens with the highest magnification had a vertical resolution of 5 nm and a lateral resolution of 10 nm. A drop of the emulsion was spread on a glass slide; this was tested, uncovered, and without further preparation. Images were recorded within 30 min after the sample preparation. An optical image of the bitumen emulsion is presented in Figure S1 in the Supporting Information (SI). Particle size distribution was analyzed by the dynamic light scattering (DLS) method using a Zetasizer Nano-ZS (Malvern Instruments Ltd., Malvern, U.K.). A He–Ne laser with λ = 632.8 nm was used for this purpose. The measurements were performed in quartz cuvettes. The DLS data were analyzed by the cumulant method. The values of the Z-average mean diameter presented in the results are the average of three replicates. A curve of the distribution of bitumen particles in the bitumen emulsion is shown in Figure S2 in the SI.

### NMR Technique

NMR spectra measurements were executed on a Bruker Avance III/Aeon 400WB (Bruker BioSpin AG) NMR spectrometer with a working frequency for protons of 400.21 MHz (induction of the static magnetic field 9.4 T). ^1^H NMR spectra were obtained by a fast Fourier transformation (FFT) following the 90° radiofrequency pulse (90°-acq.).

Pulsed gradient spin echo-nuclear magnetic resonance (PGSE-NMR) measurements were performed with a PGSE-NMR probe Diff50 (Bruker). A sample was placed in a standard 5 mm glass sample tube. The tube was closed with a plastic stopper to avoid air contact. Prior to the measurements, the sample was equilibrated at a specific temperature for 15 min. The diffusional decays (DD) were recorded using the stimulated echo (StE) pulse train. For single-component diffusion, the form of the DD can be described as^17,29^
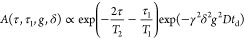
1Here, *A* is the integral intensity of the NMR signal, τ is the time interval between the first and second radiofrequency pulses, τ_1_ is the time interval between the second and third radiofrequency pulses, γ is the gyromagnetic ratio for protons, *g* and δ are the amplitude and duration of the gradient pulse, respectively, *t*_d_ = (Δ – δ/3) is the diffusion time, Δ is the time interval between two identical gradient pulses, and *D* is the diffusion coefficient. In the measurements, the duration of the 90° pulse was 7 μs, δ was in the range of 1–3 ms, τ was in the range of 3–5 ms, and *g* was varied from 0.06 up to the maximum of the gradient amplitude, 29.73 T·m^–1^. Diffusion time was in the range of 2.5–3500 ms. The repetition time during the accumulation of signal transients was 5 s. If DD is not exponential in form, an averaged apparent diffusion coefficient can be calculated from the equation:
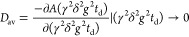
2Each experiment was repeated at least three times for each sample, and therefore, the obtained data are reproducible. Experimental errors in most of our measurements did not exceed the size of the symbols on the corresponding graph. In other cases, error bars are shown on each graph. The number of accumulations was from 32 to 1600 to provide a sufficient signal-to-noise ratio.

## Results and Discussion

**Figure 1:**
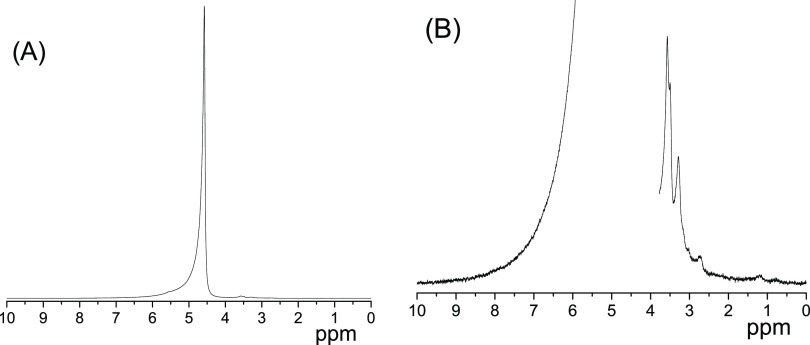
^1^H NMR spectrum of the bitumen emulsion: (A) whole range and (B) magnified part related to organic components.

The ^1^H NMR spectrum of the bitumen emulsion (Figure 1) contains one strong signal at ∼4.6 ppm, which mainly corresponds to water protons, and several signals in the range of 0–3.7 ppm, which can be related to organic (bitumen and surfactant) signals. It is possible that the dominating water signal also masks some organic signals.

**Figure 2:**
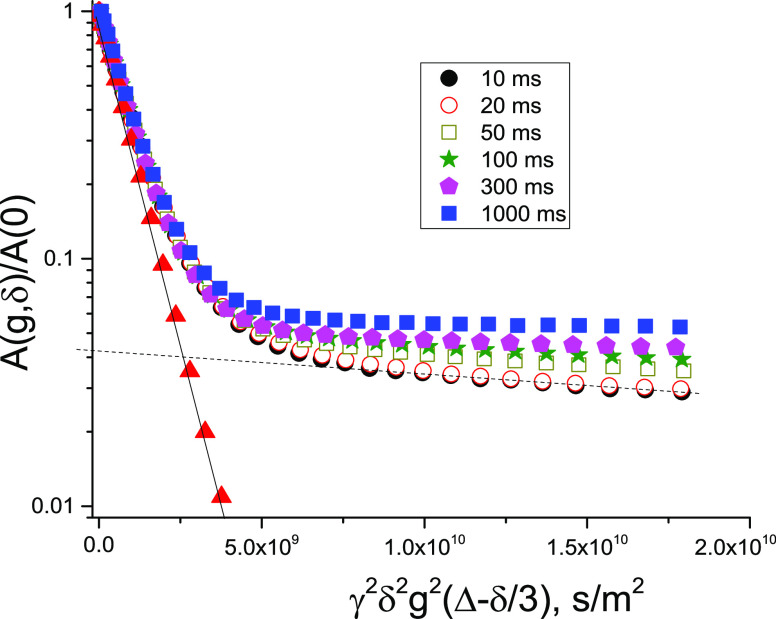
^1^H diffusion decays obtained for the bitumen emulsion at 295 K and the range of the signal decay down to 1.5 decimal orders. The duration of the gradient pulse δ varies from 0.5 ms (*t*_d_ = 10 ms) to 1 ms (*t*_d_ = 1000 ms). The maximum of the pulsed gradient amplitude varies from 5 T/m (*t*_d_ = 10 ms) to 1 T/m (*t*_d_ = 1000 ms). Fast-decaying (FDC) and slow-decaying components (SDC) of DDs are shown schematically by solid and dashed lines, respectively. Triangles correspond to the bulk water diffusion decay.

^1^H diffusion decays (DDs) obtained for the bitumen emulsion at 295 K at different diffusion times are shown in Figure 2. In the range of the signal decay (two decimal orders), each curve can be approximated by the sum of two exponentially decaying components: the fast-decaying (FDC) and the slow-decaying (SDC) components, which are shown by solid and dashed lines, respectively. The fast-decaying component gives the dominant contribution to the signal, more than 95%.

**Figure 3:**
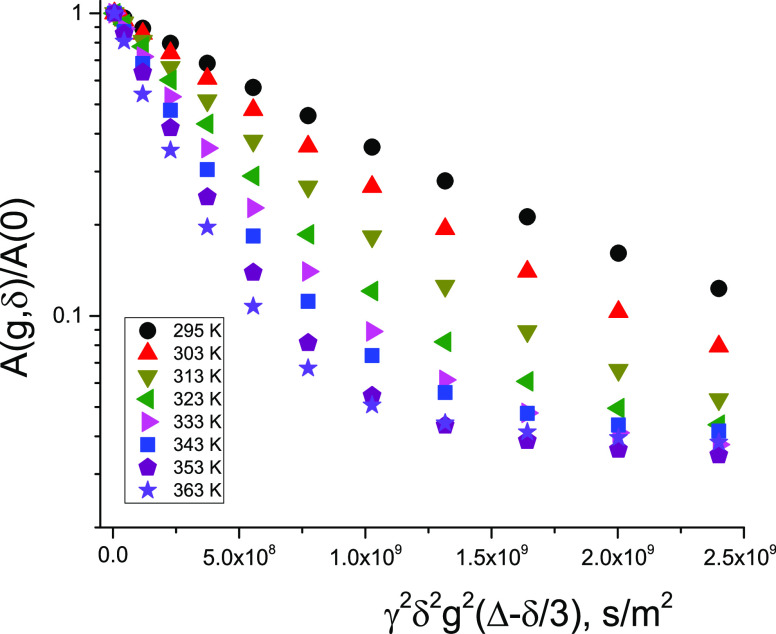
Temperature dependence of the initial part of the diffusion decay (obtained for the bitumen emulsion) corresponds to the mean diffusion coefficient in the system. Diffusion time is equal to 10 ms. The duration of the gradient pulse δ is 1 ms, and the maximum of the pulsed gradient amplitude is 5 T/m.

**Figure 4:**
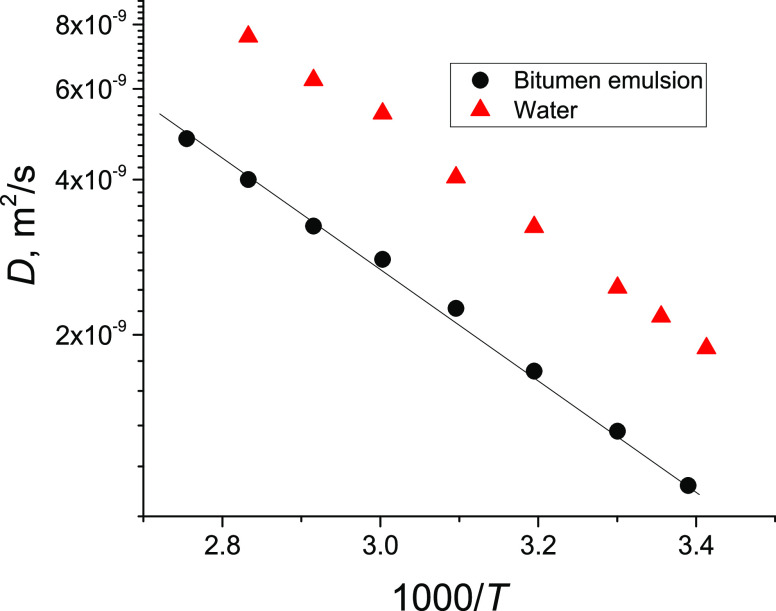
Arrhenius plots of the mean diffusion coefficient of the bitumen emulsion (black) and of neat bulk water (red) in a separate test tube obtained by ^1^H NMR.

Temperature variation of the initial parts of the DDs, which was mainly contributed by the FDC, is shown in Figure 3. As can be seen, the initial slope of the decay, which represents a mean diffusion coefficient, increases with increasing temperature. Arrhenius plots of temperature dependences of the mean diffusivities of the bitumen emulsion and water are shown in Figure 4. The mean values of the diffusion coefficients are presented in Table S1 in the SI.

The Arrhenius equation for diffusivity has the form:
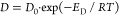
3where the slope of the dependence is determined by the apparent energy for diffusion *E*_D_ and *R* is the universal gas constant. From Figure 4, it can be seen that both dependences for the bitumen emulsion and for water have the same slope. Therefore, the mean diffusivity representing the FDC has an activation energy close to that of neat bulk water (18 kJ/mol^30^). Values of *E*_D_ estimated from the slope are ∼18.5 kJ/mol. On the other hand, the values of diffusion coefficients of the bitumen emulsion are a factor of 2 less than those of neat water. The reasons for this might be elastic collisions of water molecules with bitumen droplets and/or interactions (adsorption–desorption) of water molecules with the surface of the droplets, which might be covered by surfactants.

**Figure 5:**
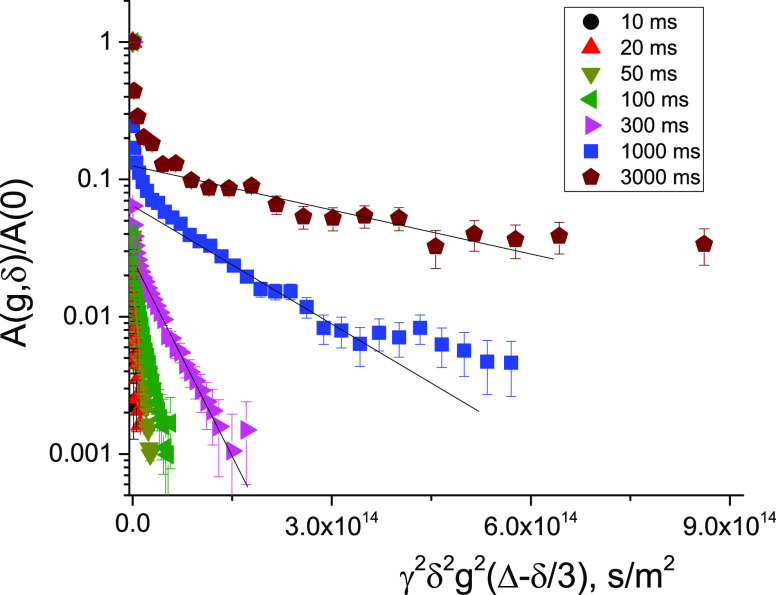
^1^H diffusion decays down to four decimal orders for the bitumen emulsion at 295 K. The duration of the gradient pulse δ is 3 ms, and the maximum of the pulsed gradient amplitude is 29.73 T/m. A signal-to-noise ratios of ^1^H NMR spectra for the different diffusion times and *g* = 0 are in Table S3 in the SI.

While the initial slopes of the DDs do not depend on the diffusion time (Figure 2), the slopes of SDC do decrease with an increase in the diffusion time. This becomes more obvious if we measure the diffusion decays at a higher degree of signal decay (Figure 5). In this presentation, information about the FDC is almost lost, while parts of DDs related to SDC at different diffusion times are more distinguishable. Measurements at longer diffusion times suggest longer time intervals in the pulse sequence used to obtain diffusion decays. Therefore, it takes to accumulate the signal longer to keep the signal-to-noise ratios high enough. Errors of measurements (error bars) in Figure 5 are larger for longer diffusion time, 1s and 3s. To be sure that these apparent oscillations on DDs are not conditioned by the “diffusion diffraction” effect,^16,20−22^ the DDs are presented as functions of the “wave vector” *q* = (2π)^−1^γδ*g* in Figure S3 in the SI. As seen, positions of the maxima and minima of the “apparent oscillations” are different for different diffusion times, as is expected for the “diffusion diffraction”.

The dependence of DDs on the diffusion time observed for the SDC shows that the slope of this part of the DDs decreases with an increase of *t*_d_, which is typical for diffusion in the presence of obstacles or confined diffusion.^17^ Indeed, for a bulk fluid without spatial barriers in the system, a mean apparent diffusion coefficient *D*_av_ = *D* corresponds to the diffusion of particles (molecules or their associates) and does not depend on the diffusion time. *D* is related to the mean squared displacement of moving particles as
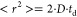
4However, if a system contains barriers, which can hinder the diffusion of the particles, the diffusion coefficient at a long enough diffusion time (estimated molecular displacements comparable to or longer than distances between barriers) is not equal to the true molecular diffusion coefficient *D*. Instead, it is named as apparent diffusion coefficient *D*_app_. *D*_app_ is smaller than *D*, and its value depends on the diffusion time, *t*_d_, the structure of the restricting geometry, and the distance between the barriers. In a particular case of fully restricted diffusion, when a liquid is confined in a droplet with impermeable walls of a characteristic size (diameter) *a*:
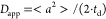
5From Figure 2, it is seen that the variation in the diffusion time does not affect the fast-decaying component represented mainly by the ^1^H NMR signal from water, according to the chemical shift at ca. 4.8 ppm (see Figure 1). This means that the effect of barriers does not change in the range of the diffusion time of the experiment and also that molecules contributing FDC do not reside under the full restriction conditions, at least up to the maximal diffusion time of the experiment, which is one second. The diffusivity may decrease relative to that of bulk water due to collisions with bitumen particles and adsorption–desorption processes on the particle’s surface, but full restriction does not occur. The lower limit of displacements can be estimated from eq 4 using *D* = 4.5 × 10^–9^ m^2^/s at a higher temperature of our measurements, 363 K, and *t*_d_ = 1 s (Figure 4). This gives an ∼95 μm.

**Figure 6:**
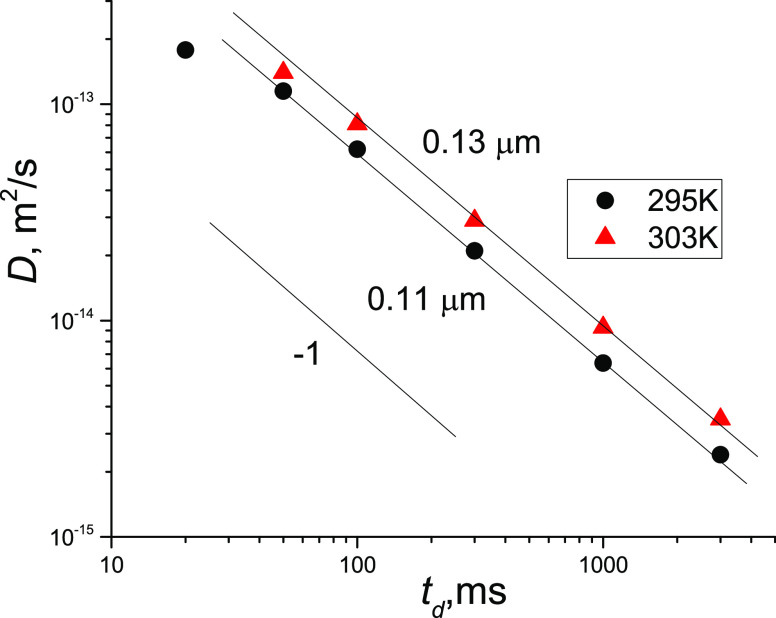
Dependences of apparent diffusion coefficients on the diffusion time for the SDC in the bitumen emulsion at two temperatures. Diffusion coefficients were calculated from high-gradient single-component parts of the SDC. The slope −1 demonstrates fully restricted diffusion. Sizes of apparent restrictions are shown. These values are not representative of the whole set because they are calculated from only a part of the SDC; this approach also does not consider diffusion of the droplet as a whole. One can refine the result after analysis of all possible deviations from the chosen model.

A similar analysis can be performed for the diffusivity of particles contributing to the SDC of diffusion decays. To estimate the effects of restrictions in the system, dependences of *D*_app_ on *t*_d_ were presented in double-logarithmic coordinates (Figure 6). The mean values of the diffusion coefficients corresponding to different diffusion times are presented in Table S2 in the SI. In the case of a restricted geometry, this presentation allows one to identify the regime of restriction. By definition, *D* is independent of the diffusion time for free diffusion. A slope from 0 to −1 of the dependence *D* on *t*_d_ is specific for the transition from free diffusion to the regime when moving particles experience restrictions with barriers.^14,17,18^ The regime of a full restriction can be recognized from the slope of the dependence, which is −1 in these coordinates, according to eq 5, and *a* ∼ (2·*D*·*t*_d_)^0.5^. For points of the curve in Figure 6, the ratio *a* ∼ (2·*D*·*t*_d_)^0.5^ is fulfilled at the diffusion time range 30–3000 ms. Therefore, diffusivity in the bitumen emulsion related to SDC corresponds to the fully restricted regime of diffusion within boundaries with sizes of ∼0.11 μm at 295 K. A slight increase in the temperature to 303 K leads to an increase in molecular displacements. This can be due to either an increase in the voids filled with water inside the bitumen droplets or an increase in the diffusion of bitumen droplets as a whole. The size of restrictions (0.11 μm) is much smaller than the sizes of bitumen droplets in the bitumen emulsion, which are in the range 0.8–35 μm (see Figures S1 and S2 in the SI).

**Figure 7:**
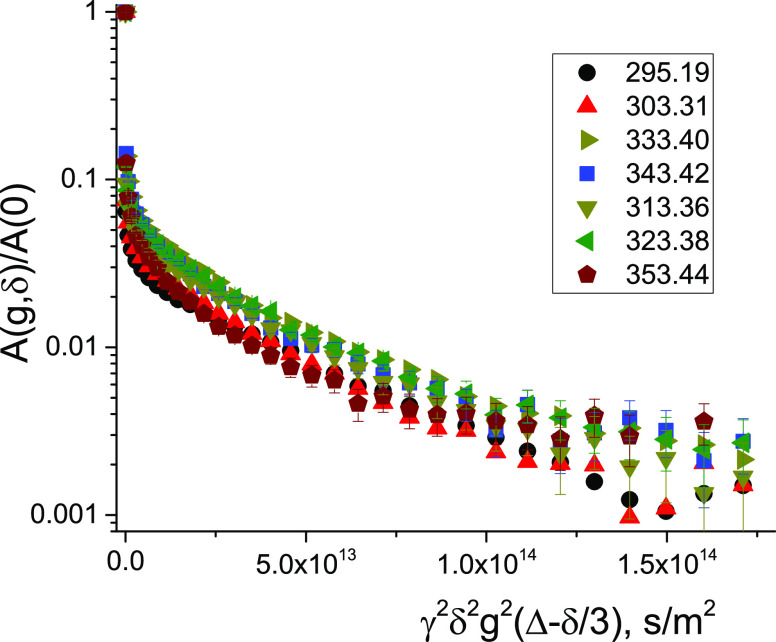
Variation of ^1^H diffusion decays for the bitumen emulsion with temperature (given by the color code in the inset). Diffusion time, *t*_d_, is equal to 300 ms. The duration of the gradient pulse δ is 3 ms, and the maximum of the pulsed gradient amplitude is 29.73 T/m. There are some changes in the form of the diffusion decays; however, these changes are minor and they are not characteristic for free, nonrestricted diffusion. For fully restricted diffusion, thermal activation of the translational molecular motion does not facilitate the particle’s displacement.

Temperature dependence of diffusivity for molecules under full restriction generally does not follow thermal activation of the translational mobility of molecules. The dependence could be defined by a thermal change in the arrangement of barriers. From Figure 7, it can be seen that slopes of DDs corresponding to SDC show almost no change at heating in the temperature range of 295–353 K. This means that the structure of the emulsion undergoes almost no change at heating in the temperature range typical for the cold mix asphalt emulsions.

The effect of fully restricted diffusion is expected for the bitumen emulsion, which contains, alongside a continuous aqueous phase, the dispersed phase of bitumen particles. Diffusion decay is generally of a complex form and depends on the ratio between the mean squared displacements (MSD) and the distance between the barriers.^19^ Therefore, the sizes of droplets and their internal structure can influence the observed form of the confinement-caused diffusion decays and their dependences on the diffusion time. The best way to describe this behavior in more detail is to validate a model of the structure of the particle of bitumen emulsion and of the droplet size distribution. Diffusion of a droplet as a whole, which is due to the thermal energy, may also contribute to the apparent diffusion coefficient in the dispersed phase.

The dispersed phase (bitumen droplets) contains bitumen and also may contain water and molecules of surfactants (emulsion stabilizers). Molecules contained inside the droplets may experience fully restricted diffusion, contributing to the SDC. To reveal which kinds of molecules experience fully restricted diffusion, let us analyze the ^1^H NMR spectra obtained at different points of the diffusion decay, corresponding to both the FDC and SDC of the diffusion decay.

**Figure 8:**
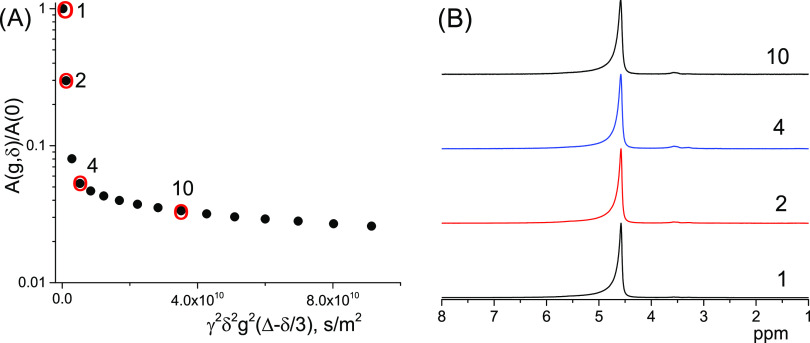
(A) ^1^H NMR diffusion decay of the bitumen emulsion. *T* = 303 K. *t*_d_ = 20 ms. (B) ^1^H NMR spectra obtained in points 1, 2, 4, and 10 of the diffusion decay in (A).

Figure 8A shows a typical two-component DD. ^1^H NMR spectra obtained at four selected points of the diffusion decay are shown in Figure 8B. Points “1” and “2” mainly correspond to the FDC, i.e., the signal decay in the continuous phase of the system (Figure 8A) where molecules do not experience fully restricted diffusion. Therefore, the signal from water is evidently dominant here, as seen in Figure 8B (spectra 1 and 2). At points “4” and “10”, the signal from the FDC, representing the continuous phase, is suppressed by the pulse-field gradient of the PFG NMR sequence (Figure 8A). Therefore, the ^1^H NMR signal at these points corresponds to the dispersed phase. It is seen in Figure 8B (spectra 4 and 10) that the signal of water protons also dominates in this case. However, relative intensities of signals from water and bitumen signals do not correspond to their real fractions because of the enhanced NMR relaxation of less-mobile molecules of bitumen in comparison with the fast orientation mobility of water. Therefore, the water signal is dominant, and the restricted diffusion of water confined inside the dispersed phase of bitumen droplets is responsible for the diffusion time dependence of diffusion decays. Generally, this demonstrates that both continuous and dispersed phases of the bitumen emulsion contain water and that the studied emulsion is a type of multiple (double) emulsion. Restricted diffusion is observed for water inside the bitumen droplets with sizes of the order of 0.11 μm that defines intradroplet areas of confined water. It should be noticed that there is no discrepancy between the data obtained from optical images and DLS (Figures S1 and S2 in the SI) and the mean size of restrictions obtained from the NMR diffusion data. Indeed, bitumen droplets are not transparent to see their internal structure. Other hand, the DLS data provide sizes of the emulsion droplets, which are in the range of 1–300 μm, while the NMR diffusion data (average size around 0.11 μm) provide data on the restriction diffusion of water inside the emulsion droplets.

**Figure 9:**
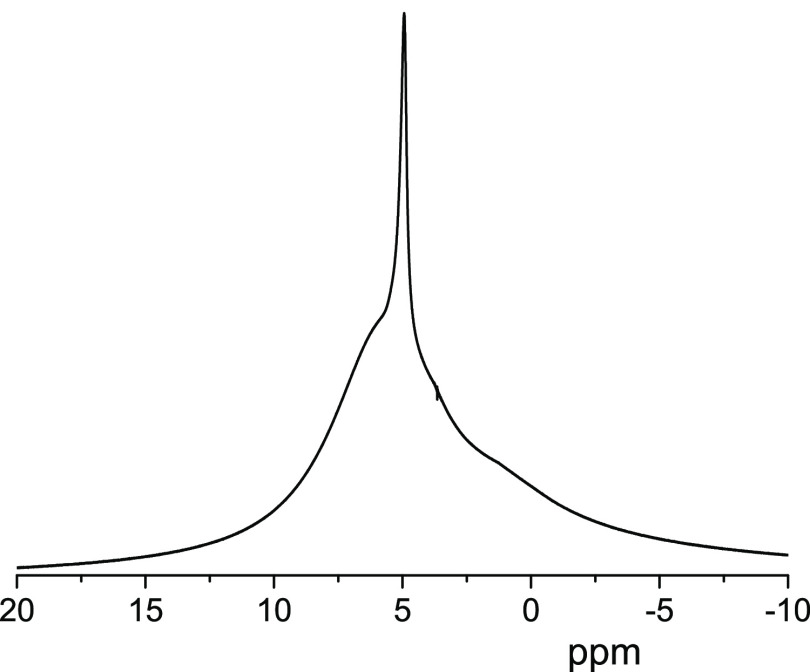
^1^H NMR spectra of the bitumen emulsion in the presence of 1.19 mM Mn^2+^(aq) ions. *T* = 295 K.

An effective method to discriminate between intradroplets and outer (continuous phase) water is the use of a doping agent, a paramagnetic salt at small enough concentrations to not interfere with the primary structure of the system.^31^ We investigated the effect of Mn^2+^(aq) ions on the ^1^H NMR spectra of the bitumen emulsion. Aqueous (Milli-Q water) solutions of Mn(II)SO_4_ with different concentrations of the salt were prepared. Then, 10 μL of the stack solution was added to 1 mL of the bitumen emulsion and gently mixed using a glass pin. The concentrations of Mn^2+^(aq) ions were calculated considering the volume of the whole mixture and were in the range of 0.12–2.38 mM. After half an hour of equilibration, the mixture was transferred to the NMR tube and analyzed by ^1^H NMR. The ^1^H NMR spectroscopy and ^1^H NMR diffusometry measurements were repeated on the same samples 2 weeks after the MnSO_4_(aq) addition to the bitumen emulsion, and the same results were obtained. The ^1^H NMR spectra showed that in the presence of Mn^2+^(aq) the signal is the sum of two overlapping components: one component presents a narrow line with a line width comparable to water in the bitumen emulsion without addition of MnSO_4_(aq). The line width of the broad component progressively increases as the concentration of paramagnetic Mn^2+^(aq) ions increases. An example of such a spectrum is shown in Figure 9. The form of the spectrum agrees well with the presence of two fractions of water in the system of the multiple emulsion. The narrow line corresponds to the intrabitumen-particle water of the dispersed phase, and its broadening is less sensitive to the presence of the paramagnetic Mn^2+^(aq) ions in the continuous (water) phase, in which bitumen particles are dispersed. The broad component corresponds to the continuous aqueous phase, which contains the added paramagnetic Mn^2+^(aq) ions. Broadening of this spectral component increases as the concentration of the paramagnetic Mn^2+^(aq) ions increases in the continuous phase.

**Figure 10:**
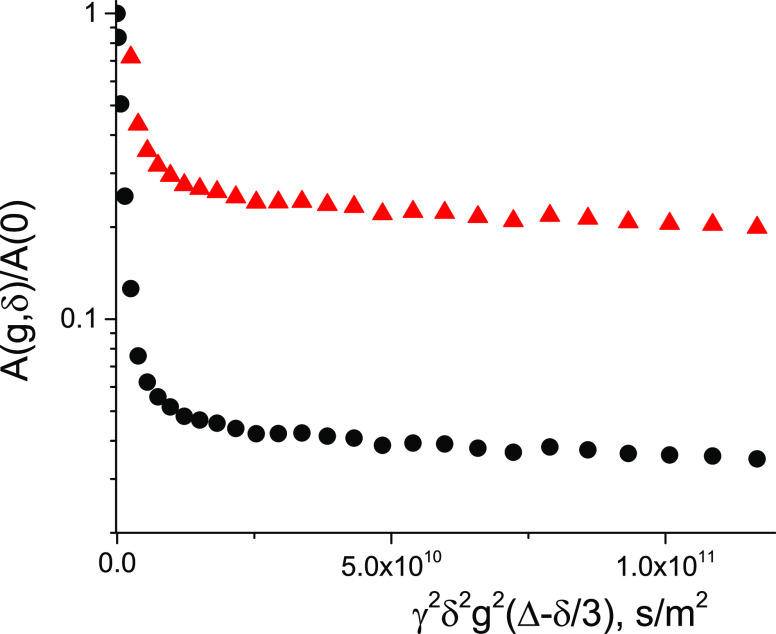
^1^H NMR diffusion decays for the bitumen emulsion without (black) and in the presence (red) of paramagnetic ions Mn^2+^(aq) at a concentration of 1.19 mM. *T* = 295 K. The diffusion time is equal to 50 ms.

An effect of the paramagnetic ions on ^1^H diffusion decays of the bitumen emulsion is shown in Figure 10. It can be seen that the FDC decreased in amplitude in the presence of paramagnetic ions, while other features of the decay remained unchanged.

An additional proof of the multiple emulsion structure of the bitumen emulsion can be obtained in a special experiment in which the original structure of the emulsion is destabilized. To destabilize the emulsion, the sample of the emulsion was placed at 253 K (−20 °C) for 12 h. After it is moved back to 295 K, the emulsion macroscopically separates into a black dense bituminous fraction at the top and a brownish aqueous fraction at the bottom of the tube. These two fractions were separated in different NMR tubes, and diffusion ^1^H NMR measurements for each of these fractions were performed.

**Figure 11:**
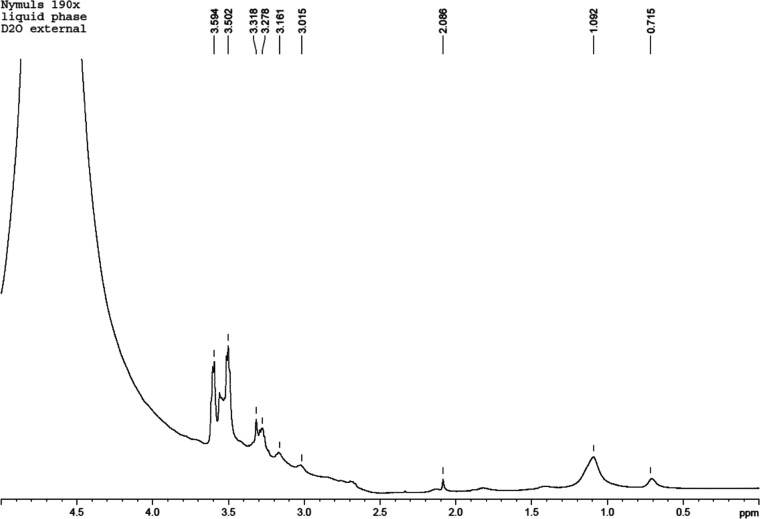
^1^H NMR spectrum of the brownish aqueous component obtained after conditioning of the bitumen emulsion at 253 K for 12 h.

The ^1^H NMR spectrum of the brownish aqueous phase is shown in Figure 11. It contains signals from water and organic components, with organic components of the sample resolved considerably better than in the original bitumen emulsion (Figure 1).

**Figure 12:**
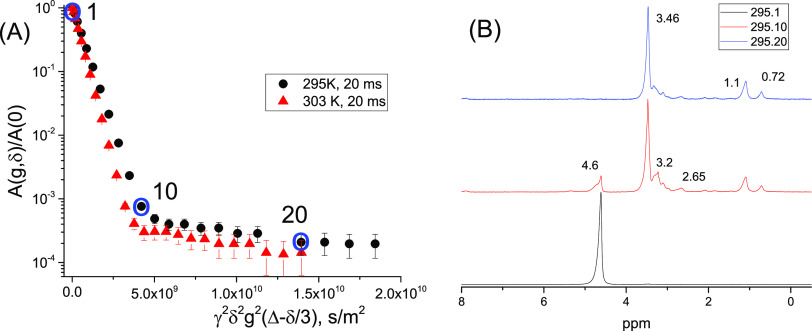
Brownish aqueous fraction of the sample of the bitumen emulsion conditioned at 253 K during 12 h. (A) ^1^H NMR diffusion decays at 295 (black) and 303 K (red). (B) Normalized ^1^H NMR spectra corresponding to points 1, 10, and 20 of the diffusion decay from bottom to top, respectively, shown by blue circles in (A). *T* = 295 K.

The DD of the aqueous component is shown in Figure 12A. It has a two-component form similar to that in the ^1^H NMR spectrum of the original emulsion (compare with Figures 2 and 5). Analysis of the FDC of the DD resulted in a diffusion coefficient *D* ∼ 2 × 10^–9^ m^2^/s, which is related to water and other small organic molecules dissolved in water. The SDC of the DD is characterized by a mean diffusion coefficient *D* ∼ 6 × 10^–11^ m^2^/s, which is a factor of ∼30 less than that of water and typical for micelles formed by surfactant molecules.^32,33^ The fraction of this component is ∼5 × 10^–4^, which is around 10^3^ less than that in the original emulsion (see Figures 2 and 5). The ^1^H NMR spectra of the aqueous component obtained at different degrees of the NMR signal suppression (points 1, 10, and “20” in Figure 12A) are shown in Figure 12B.

In normalized ^1^H NMR spectra, a dominating at point 1 signal with the chemical shift of water (4.6 ppm) has decreased dramatically at point 10 of the DD, and it is almost completely suppressed at point 20, while signals of organic molecules (0.72, 1.1, 2.65, 3.2, and 3.46 ppm) remained. This means that there is no confined water in micelles formed by organic molecules in this sample, and the aqueous phase of the sample contains micelles formed by molecules/ions of organic components (surfactants and bitumen).

**Figure 13:**
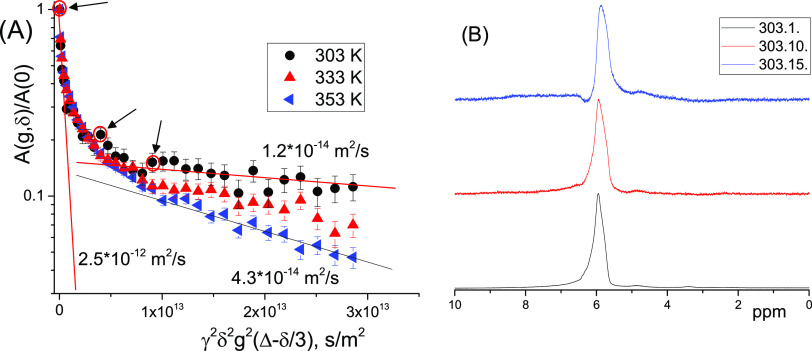
Dense bituminous fraction of the sample of bitumen emulsion conditioned at 253 K during 12 h: (A) ^1^H NMR diffusion decays at 303, 333, and 353 K. (B) Normalized ^1^H NMR spectra corresponding to points 1, 10, and “15” (from bottom to top) in the diffusion decay (A) shown by red circles and arrows. *T* = 303 K.

The ^1^H NMR spectrum of the dense bituminous part could not be obtained at 295 K because of the short NMR relaxation times of molecules with a slow orientation mobility. At temperatures of 303 K and higher (Figure 13B), the spectrum comprises a broad resonance line centered near 6 ppm with unresolved spectral features and a small, broad signal of water near 4.6 ppm. DDs for this part obtained at three different temperatures are shown in Figure 13A. These diffusion decays are of a complicated form. The very beginning part of the decays is characterized by the mean apparent diffusion coefficient of the system of ∼2.5 × 10^–12^ m^2^/s and does not change with temperature. The apparent fraction of the fast-decaying component that mainly determines the value of the mean diffusion coefficient is ∼0.85. Such highly mobile molecules may contain some small amount of residual water and light components of the bitumen. Other components of the diffusion decays corresponding to slow-moving molecules show nonsingle-exponential diffusivity. As the temperature increases, diffusivities of different components generally increase, but they change with different rates. This is a result of processes occurring in the system at heating and of an increase in the contribution of different fractions of the bitumen due to the increase in NMR relaxation times of their protons.

The ^1^H NMR spectra for three different points of the DD obtained at 303 K (Figure 13A) normalized to the maximum intensity are shown in Figure 13B. It can be seen that the form of the spectrum is maintained almost unchanged. Therefore, molecules of bitumen diffusing at different rates have identical or similar chemical structures.

## Conclusions

Self-diffusion in a bitumen emulsion was investigated by ^1^H NMR. Our study shows that NMR diffusometry can be used to characterize “multiple emulsions”. The emulsion is formed by two phases: continuous and dispersed. The continuous phase contains mainly water with an energy of activation of the diffusion process close to that of bulk water, while its diffusivity is lower than that of bulk water by a factor of 2. The dispersed phase is made up of bitumen droplets containing confined water, which is characterized as a fully restricted diffusion regime in cavities with sizes of ∼0.11 μm. Therefore, the studied bitumen emulsion is a multiple emulsion, that is, of the water/oil/water (W/O/W) type. This agrees with ^1^H NMR spectroscopy and diffusometry of the bitumen emulsion doped with paramagnetic MnSO_4_, as well as an NMR study of the emulsion structure destabilized by conditioning at 253 K during 12 h.

## Supporting Information Available

Optical image of the bitumen emulsion obtained by laser diffractometry data and of the bitumen particle size distribution obtained from DLS data (PDF)

## References

[ref1] Miljković M.; Radenberg M. Characterising the influence of bitumen emulsion on asphalt mixture performance. Mater. Struct. 2015, 48, 2195–2210. 10.1617/s11527-014-0302-y.

[ref2] Tarrer A. R.; Wagh V. In The Effect of the Physical and Chemical Characteristics of the Aggregate on Bonding, Strategic Highway Research Program (SHRP-A/UIR-91-507), National Research Council, 1991.

[ref3] Needham D. Developments in Bitumen Emulsion Mixtures for Roads. Ph.D. Thesis, University of Nottingham, 1996.

[ref4] Emulsion Formation and Stability, Tadros T. F., Ed.; Wiley-VCH Verlag GmbH & Co, 2013.

[ref5] Wong S. F.; Lim J. S.; Dol S. S. Crude oil emulsion: A review of formation, classification and stability of water-in-oil emulsions. J. Pet. Sci. Eng. 2015, 135, 498–504. 10.1016/j.petrol.2015.10.006.

[ref6] Tausk R. J. M.; Wilson P. N. Colloid chemical studies on bitumen-in-water emulsions part I. absorption of water in the bitumen droplets and other factors affecting emulsion viscosity. Colloids Surf. 1981, 2, 71–80. 10.1016/0166-6622(81)80054-6.

[ref7] Tanoura M.; Tatsuhara K.; Hiraki A.; Ogura T. Proceedings of First World Congress on Emulsion, Paris, France, 1993.

[ref8] Claudy P.; Letoffe J.-M.; Germanaud L.; Chaverot P. In Characterisation of Thermal Behaviour of Asphalts and Heavy Fuel Emulsions by DSC, 1st World Congress on Emulsion, 1993.

[ref9] Tausk R. J. M.; Wilson P. N. Colloid chemical studies on bitumen-in-water emulsions part II. Particle size analysis with a disc centrifuge. Colloids Surf. 1981, 2, 81–88. 10.1016/0166-6622(81)80055-8.

[ref10] Furlong S.; James A.; Kalinowski E.; Thompson M. Water enclosed within the droplets of bitumen emulsions and its relation to viscosity changes during storage. Colloids Surf., A 1999, 152, 147–153. 10.1016/S0927-7757(98)00628-1.

[ref11] Ding S.; Serra C. A.; Vandamme T. F.; Yu W.; Anton N. Double emulsions prepared by two–step emulsification: History, state-of -the-art and perspective. J. Controlled Release 2019, 295, 31–49. 10.1016/j.jconrel.2018.12.037. 30579983

[ref12] Dalmazzone C.; Noik C.; Clausse D. Application of DSC for emulsified system characterization. Oil Gas Sci. Technol. 2009, 64, 543–555. 10.2516/ogst:2008041.

[ref13] Neuman C. H. Spin echo of spins diffusing in a bounded medium. J. Chem. Phys. 1974, 60, 4508–4511. 10.1063/1.1680931.

[ref14] Kärger J. NMR self-diffusion studies in heterogeneous systems. Adv. Colloid Interface Sci. 1985, 23, 129–148. 10.1016/0001-8686(85)80018-X.

[ref15] Cotts R. M.; Hoch M. J. R.; Sun T.; Markert J. T. Pulsed field gradient stimulated echo methods for improved NMR diffusion measurements in heterogeneous systems. J. Magn. Reson. 1989, 83, 252–266. 10.1016/0022-2364(89)90189-3.

[ref16] Callaghan P. T.; Coy A.; MacGowan D.; Packer K. J.; Zelaya F. O. Diffraction-like effects in NMR diffusion studies of fluids in porous solids. Nature 1991, 351, 467–469. 10.1038/351467a0.

[ref17] Callaghan P. T. Principles of Nuclear Magnetic Resonance Microscopy; Clarendon Press: Oxford, 1991.

[ref18] Sen P. N. Time-dependent diffusion coefficient as a probe of geometry. Concepts Magn. Reson., Part A 2004, 23, 1–21. 10.1002/cmr.a.20017.

[ref19] Hürlimann M. D.; Helmer K. G.; de Swiet T. M.; Sen P. N.; Sotak C. H. Spin echoes in a constant gradient and in the presence of simple restriction. J. Magn. Reson., Ser. A 1995, 113, 260–264. 10.1006/jmra.1995.1091.

[ref20] Balinov B.; Jönsson B.; Linse P.; Söderman O. The NMR self-diffusion method applied to restricted diffusion. Simulation of echo attenuation from molecules in spheres and between planes. J. Magn. Reson., Ser. A 1993, 104, 17–25. 10.1006/jmra.1993.1184.

[ref21] Linse P.; Söderman O. The validity of the short-gradient-pulse approximation in NMR studies of restricted diffusion. Simulations of molecules diffusing between planes, in cylinders and spheres. J. Magn. Reson., Ser. A 1995, 116, 77–86. 10.1006/jmra.1995.1192.

[ref22] Balinov B.; Linse P.; Söderman O. Diffusion of the dispersed phase in a highly concentrated emulsion: Emulsion structure and film permeation. J. Colloid Interface Sci. 1996, 182, 539–548. 10.1006/jcis.1996.0498.

[ref23] Malmborg C.; Torgaard D.; Söderman O. NMR diffusometry and the short gradient pulse limit approximation. J. Magn. Reson. 2004, 169, 85–91. 10.1016/j.jmr.2004.04.004. 15183360

[ref24] Kärger J.; Valiullin R.; Vasenkov S. Molecular dynamics under confinement to one dimension: options of measurement and accessible information. New J. Phys. 2005, 7, 15 10.1088/1367-2630/7/1/015.

[ref25] Kortunov P.; Vasenkov S.; Kärger J.; Valiullin R.; Gottschalk P.; Fé Elía M.; Perez M.; Stöcker M.; Drescher B.; McElhiney G.; Berger C.; Gläser R.; Weitkamp J. The role of mesopores in intracrystalline transport in USY zeolite: PFG NMR diffusion study on various length scales. J. Am. Chem. Soc. 2005, 127, 13055–13059. 10.1021/ja053134r. 16159301

[ref26] Hollingsworth K. G.; Johns M. L. Measurement of emulsion droplet sizes using PFG NMR and regularization methods. J. Colloid Interface Sci. 2003, 258, 383–389. 10.1016/S0021-9797(02)00131-5. 12618109

[ref27] Jiang T.; Hirasaki G.; Miller C.; et al. Diluted bitumen water-in-oil emulsion stability and characterization by nuclear magnetic resonance (NMR) measurements. Energy Fuels 2007, 21, 1325–1336. 10.1021/ef0604487.

[ref28] Marques D. S.; Sorland G.; Less S.; Vilagines R. The application of pulse field gradient (PFG) NMR method to characterize the efficiency of separation of water-in-crude oil emulsions. J. Colloid Interface Sci. 2018, 512, 361–368. 10.1016/j.jcis.2017.10.075. 29080532

[ref29] Tanner J. E. Use of the stimulated echo in NMR diffusion studies. J. Chem. Phys. 1970, 52, 2523–2526. 10.1063/1.1673336.

[ref30] Piskulich Z. A.; Mesele O. O.; Thompson W. H. Removing the barrier to the calculation of activation energies: diffusion coefficients and reorientation times in liquid water. J. Chem. Phys. 2017, 147, 134103 10.1063/1.4997723. 28987106

[ref31] Elsayed M.; Isah A.; Hiba M.; Hassan A.; Al-Garadi K.; Mahmoud M.; El-Husseiny A.; Radwan A. E. A review on the application of nuclear magnetic resonance (NMR) in the oil and gas industry: laboratory and field-scale measurements. J. Pet. Explor. Prod. Technol. 2022, 12, 2747–2784. 10.1007/s13202-022-01476-3.

[ref32] Söderman O.; Stilbs P. NMR studies of complex surfactant systems. Prog. Nucl. Magn. Reson. Spectrosc. 1994, 26, 445–482. 10.1016/0079-6565(94)80013-8.

[ref33] Arkhipov V. P.; Arkhipov R. V.; Kuzina N. A.; Filippov A. Study of the premicellar state in aqueous solutions of sodium dodecyl sulfate by NMR diffusion. Magn. Reson. Chem. 2021, 59, 1126–1133. 10.1002/mrc.5165. 33864285

