# Supplementary material for: Dynamics and Structure of a Bitumen Emulsion as Studied by 1H NMR Diffusometry
Source: ACS Omega. 2023 Sep 21;8(39):36534–42. doi: 10.1021/acsomega.3c05492 (PMC10552106; doi:10.1021/acsomega.3c05492)
Supplement: Supplementary file 1 — ao3c05492_si_001.pdf [file ao3c05492_si_001.pdf]

Supporting Information

Dynamics and structure of a bitumen emulsion as studied by ^{1}H NMR diffusometry

Andrei Filippov,[a]* Hilde Soenen,[b] Johan Blom,[c] Oleg N. Antzutkin[a]

[a] Chemistry of Interfaces, Department of Civil and Environmental Engineering, Luleå University of Technology, Luleå SE-97187, Sweden

[b] Nynas N.V., 171 Groenenborgerlaan, Antwerp 2020, Belgium

[c] Faculty of Applied Engineering, EMIB-research Group, University of Antwerp, 171 Groenenborgerlaan, Antwerp 2020, Belgium

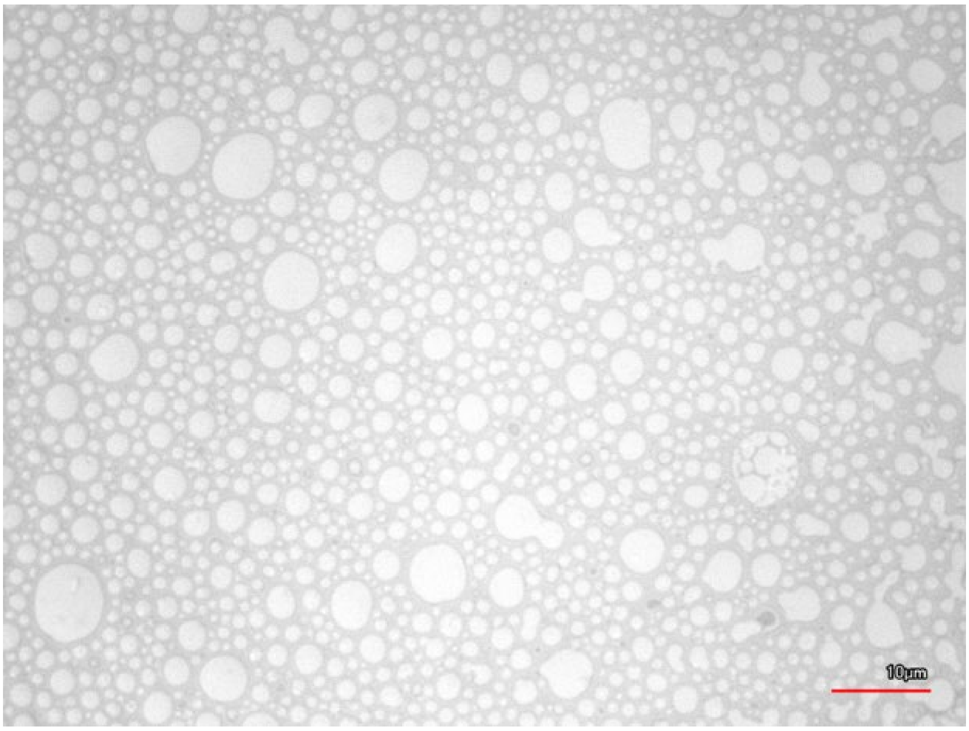

Figure S1. Optical image of the bitumen emulsion obtained by Confocal Laser Scanning microscopy

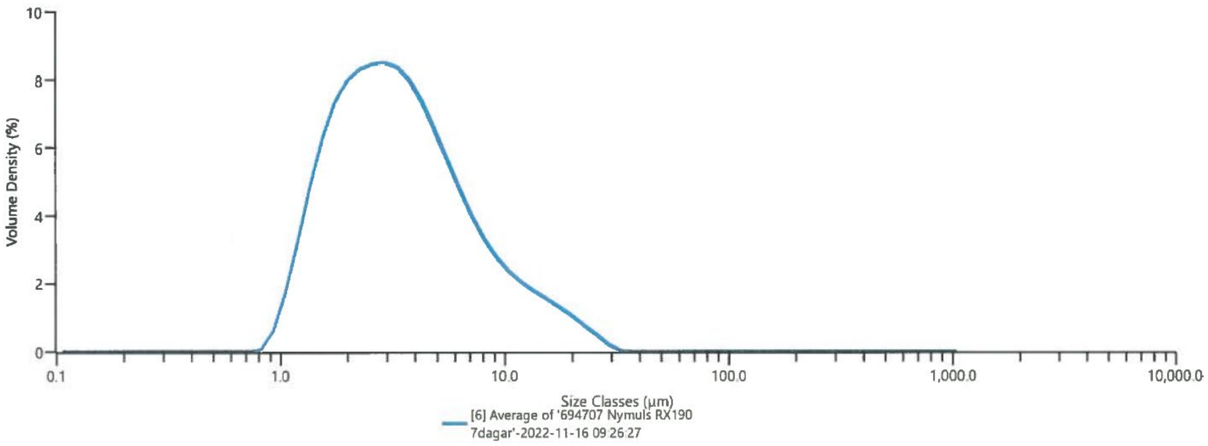

Figure S2. Bitumen particles size distribution obtained from Dynamic Light Scattering (DLS) data.

Table S1. Values of mean diffusion coefficient corresponding to the fast-decaying component (FDC) of diffusion decays in the bitumen emulsion.

T, K	295	303	313	323	333	343	353	363
$\boldsymbol{D} \times 10^{-9}$ m^2/s	1.02	1.3	1.7	2.25	2.8	3.25	4	4.8

Table S2. Values of mean diffusion coefficient (m^2/s) corresponding to the slowly decaying component (SDC) of diffusion decays in the bitumen emulsion at different diffusion times and two temperatures.

t_d, ms	20	50	100	300	1000	3000
295 K	$1.78 \cdot 10^{-13}$	$1.15 \cdot 10^{-13}$	$6.2 \cdot 10^{-14}$	$2.1 \cdot 10^{-14}$	$6.36 \cdot 10^{-15}$	$2.4 \cdot 10^{-15}$
303 K		$1.4 \cdot 10^{-13}$	$8.1 \cdot 10^{-14}$	$2.9 \cdot 10^{-14}$	$9.3 \cdot 10^{-15}$	$3.5 \cdot 10^{-15}$

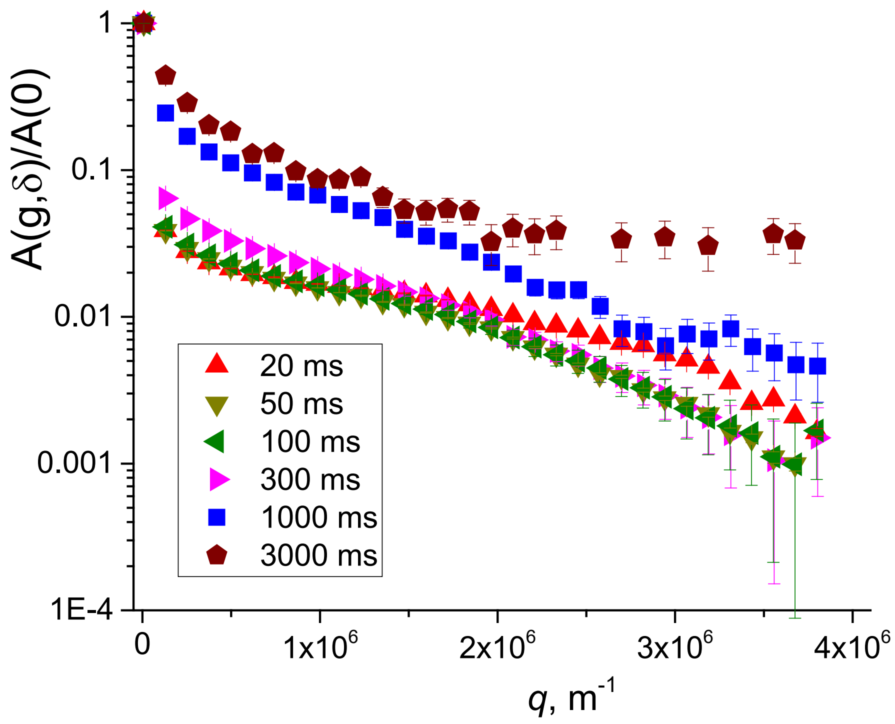

Figure S3. [1]H diffusion decays of Figure 5 in the "q-presentation". $q = (2\pi)^{-1}\gamma\delta g$. $T = 295$ K. The duration of the gradient pulse δ is 3 ms and the maximum of the pulsed gradient amplitude is 29.73 T/m.

Table S3. Signal-to-noise ratios for ^{1}H spectra at g=0 and different diffusion times for DDs presented in Figure 5.

t_d, ms	10	20	50	100	300	1000	3000
SNR	10^3	10^4	10^4	10^4	10^4	500	100
